# Autogynephilia in Some Bisexual Cisgender Men

**DOI:** 10.1007/s10508-025-03166-7

**Published:** 2025-07-02

**Authors:** James S. Morandini, Kevin J. Hsu, S. Rudd

**Affiliations:** 1 School of Psychology, University of Sydney, Sydney, NSW 2006 Australia; 2 Department of Psychological and Social Sciences, Pennsylvania State University, Abington, Abington, PA USA; 3 Department of Psychology, Washington State University Vancouver, Vancouver, WA USA

**Keywords:** Autogynephilic, Paraphilia, Bisexuality, Sexual orientation, Gender dysphoria, Autogynephilic bisexuality

## Abstract

Although some bisexual men experience conventional sexual attraction to both males and females, others have less obvious sexual interests underlying their bisexual identity, attraction, or behavior (i.e., bisexual phenomena). Accordingly, it has been proposed that some men’s bisexual phenomena are motivated by autogynephilia, a male’s propensity to be sexually aroused by the thought or image of being a woman. For these men, sexual attraction to males is secondary to their autogynephilia, rather than a reflection of conventional attraction to males. The present study examined how common autogynephilia is among bisexual cisgender men, and how autogynephilic bisexual men may differ from non-autogynephilic bisexual men in their sexual interests and behavior, uncertainty about their sexual orientation, and gender-related experiences. We recruited a large sample of bisexual-identified men (*n* = 254) via Prolific to complete an online survey. Of the sample, 35% (*n* = 89) endorsed at least half of the items on a measure of core autogynephilia. Consistent with past research on autogynephilia, autogynephilic bisexual men reported more paraphilic sexual interests than non-autogynephilic bisexual men. Of clinical importance, autogynephilic bisexual men reported less certainty about their sexual orientation, elevated childhood and adult gender nonconformity, and elevated gender dysphoria relative to non-autogynephilic bisexuals. These findings suggest that a substantial minority of bisexual men experience autogynephilia, and that future research should consider the unique experiences and needs of autogynephilic bisexual men, including how else they might differ from other bisexual men.

## Introduction

Emerging research suggests that cisgender men (hereafter referred to as “men”) who identify as bisexual are a heterogeneous group. While some are bisexual in the conventional sense—experiencing substantial sexual attraction to both males and females (Jabbour et al., 2020; Rosenthal et al., 2012; Slettevold et al., 2019)—others display bisexual identity, attraction, or behavior for other reasons (Bailey & Rosenthal, 2015; Hsu et al., 2015, 2016; Rosenthal et al., 2017). The present study examined the understudied subgroup of bisexual men whose bisexual identity, attraction, or behavior is secondary to autogynephilia, which is a male’s propensity for sexual arousal by the thought or image of himself as a woman (Blanchard, 1989a), and how autogynephilic bisexual men differ from non-autogynephilic bisexual men.

Autogynephilia can be conceptualized as an internalization of the most common, externally directed male sexual orientation, which is heterosexual attraction to females (Blanchard, 1991; Freund & Blanchard, 1993; Hsu & Bailey, 2022; Lawrence, 2009). This internalization is not often complete, however, resulting in most autogynephilic males retaining their attraction to women while also experiencing sexual arousal by the thought or image of being a woman. It is thought that while autogynephilic bisexual men also retain their attraction to females, they do not possess conventional attraction to males like in other bisexual men (Blanchard, 1989b; Hsu et al., 2015; Lawrence, 2013). Rather, fantasizing about and sexually interacting with men can be arousing because it enhances the interpersonal aspects of their autogynephilic fantasy of being a woman. Thinking about and actually having sex with men might only be appealing when they are cross-dressing in women’s clothes or otherwise being treated as a woman.

Based on the rate of erotic cross-dressing, which is the most common manifestation of autogynephilia (Blanchard, 1991; Lawrence, 2013), it has been estimated that up to 3% of men are autogynephilic (Långström & Zucker, 2005). Recent studies of autogynephilic men have also found that a substantial minority either identify as bisexual or report bisexual attraction or behavior (Brown et al., 2020; Hsu et al., 2015). Thus, it seems that autogynephilic bisexuals may comprise a significant proportion of bisexual men. Examining the prevalence of autogynephilia in contemporary samples of bisexual men and understanding how autogynephilic and non-autogynephilic bisexual men differ, is important to research and practice with bisexual men, who are a heterogeneous group with differing concerns and needs.

### Paths to Bisexuality in Cisgender Men

Almost all men who identify as gay or heterosexual are much more sexually aroused (i.e., experience measurably greater genital arousal) by sexual stimuli involving men versus women, respectively (Bailey, 2009; Chivers et al., 2004). As such, in addition to their shared identity label, almost all gay men are preferentially sexually aroused by men, and almost all heterosexual men are preferentially sexually aroused by women. These gender-specific sexual arousal patterns appear largely inborn, are remarkably stable, and predate these men’s identification as gay or heterosexual (see Bailey et al., 2016 for a review). In fact, Bailey (2009) goes so far as to argue that a man’s sexual arousal pattern *is* his sexual orientation, and that it is the subjective awareness of his sexual arousal pattern that typically informs a man of his sexual orientation. For most gay and heterosexual men, therefore, their sexual identity (as gay or heterosexual) relates straightforwardly to their underlying erotic preference for men versus women.

It is not known what proportion of bisexual men have bisexual arousal patterns that reflect conventional sexual attraction to both males and females, or an underlying bisexual orientation as it is conventionally understood. Despite early attempts to study bisexual men that did not find evidence of bisexual arousal patterns (e.g., Rieger et al., 2005), research has since established that some bisexual men have quite straightforward bisexual arousal patterns (Jabbour et al., 2020; Rosenthal et al., 2012; Slettevold et al., 2019). That is, they are substantially more sexually aroused by women than gay men, and substantially more sexually aroused by men than heterosexual men. These findings related to sexual arousal patterns have been observed in self-report studies, as well as studies examining physiological indices of sexual arousal as diverse as genital arousal (Jabbour et al., 2020; Rosenthal et al., 2012; Slettevold et al., 2019), eye-tracking (Morandini et al., 2020), pupil dilation (Attard-Johnson et al., 2017; Rieger & Savin-Williams, 2012), viewing time (Lippa, 2013), and functional magnetic resonance imaging of neural responses (Safron et al., 2017).

Besides autogynephilia, other reasons for bisexual identity, attraction, or behavior have been studied. For example, some men who only experience sexual feelings for men will identify as bisexual. In fact, it is quite common for men to temporarily identify as bisexual before coming out as gay, a phenomenon that has been called transitional bisexuality (Greene & Croom, 2000; Guittar, 2013). A study by Semon et al. (2017) reported that gay men with a history of transitional bisexuality demonstrated sexual arousal patterns indistinguishable from gay men who did not have such a history, and more than 80% of these men believed in retrospect they had never truly been bisexual. There are also several studies that report rates as high as 50% for bisexual identification among men with gynandromorphophilia, which is a specific sexual interest in birth-assigned males who have both male and female physical characteristics, such as transgender women who have breasts while retaining a penis (Hsu et al., 2016; Operario et al., 2008, 2011; Rosenthal et al., 2017; Weinberg & Williams, 2010). In contrast to their self-reported identity, bisexual men with gynandromorphophilia did not show bisexual genital arousal patterns in the sense of having a substantial genital response to both cisgender male and female sexual stimuli (Hsu et al., 2016). It might be that gynandromorphophilic men who identify as bisexual are interpreting their sexual interest in both male and female physical features within the same person (e.g., a transgender woman with breasts and a penis) as evidence that they are bisexual, even when they do not experience sexual attraction to men outside of this context. As is the case with conventionally understood bisexuality that corresponds with a bisexual arousal pattern, it is not known what proportion of bisexual men are represented by transitional bisexuality or gynandromorphophilia. Furthermore, we do not have clear scientific consensus on how many subtypes of bisexuality exist in men, which is an important goal because different reasons for bisexuality suggest different approaches to research and clinical practice.

### Bisexual Phenomena in Autogynephilic Cisgender Men

In his early work on autogynephilia, Blanchard (1989b) coined the term pseudobisexuality to describe sexual fantasies about or sexual encounters with men that appeared to be motivated by autogynephilia, rather than an unambiguous and straightforward sexual attraction to both male and female bodies that is conventionally understood as bisexuality. Elaborating further, Blanchard (1989a) wrote that autogynephilia “may find expression in the fantasy of having intercourse, as a woman, with a man” (p. 323). To clarify the appeal of the male partner for an autogynephilic man, Blanchard (1989a) wrote: “The effective erotic stimulus in these interactions, however, is not the male physique of the partner, as it is in true homosexual attraction, but rather the thought of being a woman, which is symbolized in the fantasy of being penetrated by a man. For these persons, the male sexual partner serves the same function as women's apparel or makeup, namely, to aid and intensify the fantasy of being a woman” (pp. 323–324). Notwithstanding the accuracy of Blanchard’s observations, the term pseudobisexuality may be perceived as dismissive or invalidating of this type of bisexuality by some community members (Serano, 2020). In this paper, we therefore avoid this terminology and instead refer to this type of bisexuality as autogynephilic bisexuality and those experiencing it as autogynephilic bisexual men.

Studies find that autogynephilic men are much more likely than non-autogynephilic men to report a bisexual identity. Brown et al. (2020) found that among 522 birth-assigned males (96.7% of whom were cisgender men) reporting sexual arousal by the thought or fantasy of acting and/or dressing like a woman, 62.8% identified as heterosexual, 30.3% as bisexual, 1.5% as homosexual, 0.2% as asexual, and 5.2% as other. Hsu et al. (2015), based on a sample of 149 autogynephilic men recruited from relevant online communities, found that 80.5% identified as heterosexual, 14.7% as bisexual, and 4.8% as another sexual identity. Although bisexual phenomena are clearly prevalent in autogynephilic men, no data exist on the prevalence of autogynephilia in bisexual men.

Other than autogynephilia, how else might autogynephilic and non-autogynephilic bisexual men differ? Little has been written about men whose bisexual identity, attraction, or behavior is secondary to autogynephilia. Online communities on Reddit (e.g., r/bisexual, r/askAGP, r/sissyology) provide some clues, however. Many autogynephilic bisexual men have provided rich descriptions of their experiences in these online communities, suggesting that they may experience more confusion about their sexuality than non-autogynephilic bisexual men, possibly due to a lack of attraction to the male physique and feelings of attraction to men only when fantasizing about being a woman. For instance, a post in r/AskMenAdvice titled “Straight but Have Submissive Fantasies with Men. Am I Bisexual, bicurious, heteroromantic-heteroflexible, heteroromantic-bisexual?” (Infinity100b, 2025) details such confusion: “The strange part is that I don’t feel any real-life attraction to men. I don’t look at a guy and think, ‘I want to have sex with him.’ I don’t watch gay porn, and I never imagine myself as a man being dominated in a gay scene. Instead, my fantasies always place me in the role of a woman—whether in vanilla one-on-one or gangbang scenarios, I imagine myself as the woman being dominated.” As a result of being sexually attracted to men in this more indirect manner, autogynephilic bisexual men may also be less likely than other bisexual men to have actual sexual encounters or romantic relationships with men. Moreover, autogynephilia is known to be related to other types of sexual interests. Relative to non-autogynephilic men, autogynephilic men are more likely to experience gynandromorphophilia (Hsu et al., 2017), additional paraphilias (e.g., sexual masochism; Hsu et al., 2015), and analloeroticism (i.e., less attraction to other people; Lawrence, 2013). Autogynephilic bisexual men might likewise be elevated on these traits relative to non-autogynephilic bisexual men.

Finally, a subset of autogynephilic birth-assigned males develop gender dysphoria (i.e., discomfort or distress with the male social role and/or male anatomy, possibly emanating from a strong desire for the female social role and/or female anatomy) (Blanchard, 1989a; Brown et al., 2020; Hsu et al., 2015; Lawrence, 2009, 2013). This form of gender dysphoria may be intermittent or persistent, and its course may remain stable, remit, or intensify over time. As a means to alleviate gender dysphoria, some subsequently seek to transition medically to feminize their bodies and may transition socially to live as a woman or non-binary feminine person. It is expected that autogynephilic bisexual men would demonstrate much elevated risk of gender dysphoria than non-autogynephilic bisexual men. They are also expected to report more gender nonconformity in adulthood and possibly childhood, given their autogynephilic desire to look, dress, or act like a woman. One study found that self-reported childhood gender nonconformity was related to higher levels of autogynephilia (Brown et al., 2020).

### The Present Study

The present study examined how common autogynephilia is among bisexual men, and how autogynephilic bisexual men may differ from non-autogynephilic bisexual men in their sexual interests and behavior, uncertainty about their sexual orientation, and gender-related experiences. Compared to non-autogynephilic bisexual men, we hypothesized the following about autogynephilic bisexual men:

1. Autogynephilic bisexual men will report more frequent cross-dressing in the past 12 months, because cross-dressing is a common manifestation of autogynephilia (Blanchard, 1991; Brown et al., 2020; Hsu et al., 2015; Lawrence, 2013).
2. Autogynephilic bisexual men will report cross-dressing as more important to sexual fantasy or masturbation and sex, given the centrality of autogynephilia to the sexuality of those who experience it (Hsu et al., 2025; Lawrence, 2007).
3. Autogynephilic bisexual men will report less sexual attraction to cisgender men, and fewer sexual or romantic cisgender male partners (Blanchard, 1989b).
4. Autogynephilic bisexual men will report more sexual attraction to transgender women with a penis and to male cross-dressers, given the association between autogynephilia and gynandromorphophilia (Hsu et al., 2016, 2017; Rosenthal et al., 2017).
5. Autogynephilic bisexual men will report more paraphilic sexual interests, given the association between autogynephilia and other paraphilic interests like sexual masochism (Hsu et al., 2015).
6. Autogynephilic bisexual men will report less sexual interest in other people (Lawrence, 2013).
7. Autogynephilic bisexual men will report greater uncertainty regarding their sexual orientation, due to the possibility that their bisexuality is secondary to autogynephilia, and because they may question if they are truly attracted to men (Blanchard, 1989b).
8. Autogynephilic bisexual men will report increased symptoms of gender dysphoria in the past 12 months, and a greater likelihood of having ever experienced gender dysphoria, given the association between autogynephilia and gender dysphoria in males (Blanchard, 1989a; Brown et al., 2020; Hsu et al., 2015; Lawrence, 2009, 2013).
9. Autogynephilic bisexual men will report greater perceived childhood and adult gender nonconformity, due to their interest in being or presenting as a woman (Brown et al., 2020).

## Method

### Participants

We recruited a sample of 288 bisexual-identified cisgender men via advertisements placed on Prolific (https://prolific.co). Prolific is an online platform that connects researchers, particularly those in academia and commercial sectors, with participants for research studies. Advertisements specified that participants were required to be cisgender men and at least 18 years of age, as well as to identify as bisexual and not as cross-dressers or transfeminine. After screening out participants who were ineligible based on inclusion criteria, 254 participants met eligibility and completed the survey in its entirety.

All 254 participants reported that they were cisgender men (i.e., assigned male at birth), at least 18 years of age, bisexual, and not either a cross-dresser or transfeminine. Participants completed an anonymous online survey that was estimated to take about 60 min and hosted on Qualtrics. Participants were reimbursed $10.00 USD.

**Fig. 1:**
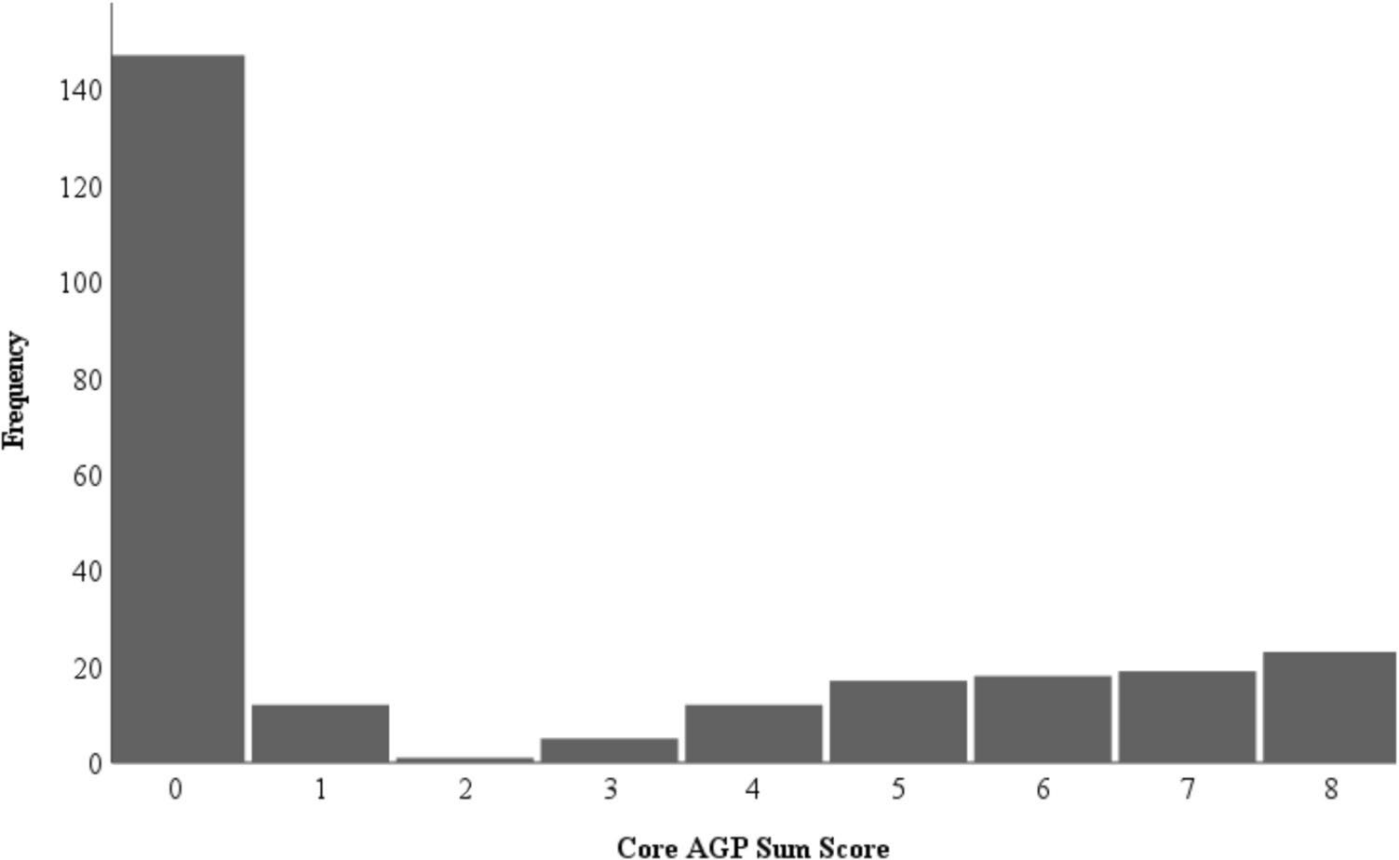
Frequency plot of scores on the Core Autogynephilia Scale (Blanchard, 1989b) among the bisexual cisgender men (*n* = 254). *Note*. Zero represents no endorsement of items reflecting autogynephilia. Eight is the maximum possible summed score. Participants with scores from 0 to 3 were categorized as non-autogynephilic bisexual men, while those with responses from 4 to 8 were categorized as autogynephilic bisexual men

The presence or absence of autogynephilia in the bisexual male sample was determined by performing a midpoint split of the Core Autogynephilia Scale (Blanchard, 1989b). Participants with scores from 0 (minimum) to 3 were categorized as non-autogynephilic, while those with responses from 4 to 8 (maximum) were categorized as autogynephilic. In the absence of an established cutoff value for the presence of autogynephilia and considering the bimodal distribution of Core Autogynephilia Scale scores in our sample (see Fig. 1), a midpoint split was deemed appropriate. Two groups of bisexual men were created: a non-autogynephilic group (*n* = 165) and an autogynephilic group (*n* = 89).

Demographics for non-autogynephilic bisexual men (*n* = 165) and autogynephilic bisexual men (*n* = 89) are reported in Table 1. No significant differences between the non-autogynephilic and autogynephilic groups were observed with regard to age (*M* age = 33.1 years; *SD* = 12.0 versus *M* age = 34.9 years; *SD* = 13.4), country of residence (57.6% versus 58.4% living in the USA), race/ethnicity (73.9% versus 73.0% White/Caucasian), romantic relationship status (49.1% versus 37.9% single), educational attainment (56.3% versus 56.2% with at least a bachelor’s degree), political orientation (76.4% versus 71.0% somewhat to very liberal, 12.7% versus 18.0% middle of the road, and 7.9% versus 11.2% somewhat to very conservative), religiosity (62.4% versus 51.7% not at all religious), and knowledge of autogynephilia as a concept (13.9% versus 18.0% had heard of it).

**Table 1:** Demographic characteristics of non-autogynephilic and autogynephilic bisexual cisgender men

Measure	Non-AGP bisexuals (*n* = 165)	AGP bisexuals (*n* = 89)
Age in years, *M* (SD)	33.2 (12.0)	34.9 (13.4)
US nationality, % (*n*)	57.6% (95)	58.4% (52)
*Race/ethnicity*		
White/Caucasian, % (*n*)	73.9% (122)	73.0% (65)
Black/African American, % (*n*)	19.4% (32)	13.5% (12)
Hispanic or Latino/a/x, % (*n*)	11.5% (19)	12.4% (11)
East Asian/Pacific Islander, % (*n*)	2.4% (4)	2.2% (2)
South Asian, % (*n*)	0.6% (1)	1.1% (1)
Arabic/Middle Eastern, % (*n*)	1.2% (2)	2.2% (2)
Native American, % (*n*)	0.6% (1)	1.1% (1)
Other, % (*n*)	1.8% (3)	2.2% (2)
*Romantic relationship status*^†^		
Single, % (*n*)	49.1% (80)	37.9% (33)
In a relationship, % (*n*)	27.0% (44)	29.9% (26)
Engaged, % (*n*)	1.2% (2)	1.1% (1)
Married, % (*n*)	20.9% (34)	28.7% (25)
Divorced, % (*n*)	1.8% (3)	2.3% (2)
Widowed, % (*n*)	0.0% (0)	0.0% (0)
*Education*		
Less than high school, % (*n*)	0.0% (0)	0.0% (0)
Some high school, % (*n*)	2.4% (4)	2.2% (2)
High school/GED, % (*n*)	17.6% (29)	15.7% (14)
Some college, % (*n*)	23.6% (39)	25.8% (23)
Bachelor’s degree, % (*n*)	47.9% (79)	38.2% (34)
Master’s degree, % (*n*)	6.6% (11)	16.9% (15)
Doctoral degree, % (*n*)	1.2% (2)	0.0% (0)
Professional degree, % (*n*)	0.6% (1)	1.1% (1)
*Political orientation*		
Very liberal, % (*n*)	29.7% (49)	24.7% (22)
Liberal, % (*n*)	29.1% (48)	27.0% (24)
Somewhat liberal, % (*n*)	17.6% (29)	19.1% (17)
Middle of the road, % (*n*)	12.7% (21)	18.0% (16)
Somewhat conservative, % (*n*)	7.9% (13)	4.5% (4)
Conservative, % (*n*)	3.0% (5)	4.5% (4)
Very conservative, % (*n*)	0.0% (0)	2.3% (2)
*Religiosity*		
Not at all religious, % (n)	62.4% (103)	51.7% (46)
A little to Extremely Religious, % (*n*)	37.6% (62)	48.3% (43)
*Knowledge of autogynephilia*		
Yes, % (*n*)	13.9% (23)	18.0% (16)
No, % (*n*)	79.4% (131)	76.4% (68)
Unsure, % (*n*)	6.7% (11)	5.6% (5)

^†^Two participants in each group did not register a response to the relationship status item

### Measures

Bisexual participants completed demographic questions and self-report measures of sexual interests and behavior, uncertainty about their sexual orientation, and gender-related experiences.

#### Demographics

Demographics collected include age, country of residence, sex assigned at birth, gender identity, sexual orientation, race/ethnicity, romantic relationship status, educational attainment, political orientation, religiosity, and knowledge of autogynephilia as a concept. For sex assigned at birth, participants were asked to choose from male, female, or other. For gender identity, participants were asked what gender they identify, feel, present, or live most of the time and to choose one of the following categories: male/man, female/woman, transfeminine/trans woman, transmasculine/trans man, non-binary/enby, genderfluid, questioning, agender, and other. For sexual orientation, participants were asked what sexual orientation they identify, feel, present, or live most of the time and to choose one of the following labels: heterosexual/straight, bisexual, gay/lesbian, asexual, pansexual, and other. No participants selected a sex assigned at birth that was not male, a gender identity that was not male/man, or a sexual orientation that was not bisexual, confirming that all participants in our sample met the inclusion criteria and that prior screening procedures in Prolific were successful.

For race/ethnicity, participants were asked to choose one or more of the following options: White/Caucasian, Black/African American, Hispanic or Latino/a/x, East Asian/Pacific Islander, South Asian, Arabic/Middle Eastern, Native American, and other race/ethnicity. Because participants could choose more than one option for race/ethnicity, the percentages in Table 1 do not add up to 100%. For romantic relationship status, participants were asked to choose from one of the following options: single, in a relationship, engaged, married, divorced, and widowed. For educational attainment, participants were asked to choose from one of the following options: less than high school, some high school, high school/GED, some college, bachelor’s degree, master’s degree, doctoral degree, and professional degree.

For political orientation, we asked participants to respond to the question “Which of the following best describes your current political orientation?” on a seven-point scale from 1 (*very liberal*) to 7 (*very conservative*). For religiosity, we asked participants to respond to the question “How religious would you consider yourself currently?” on a seven-point scale from 1 (*not at all*) to 7 (*extremely*). For knowledge of autogynephilia as a concept, we asked participants to respond to the question “Have you ever heard of the term autogynephilia?” with the options of “yes,” “no,” or “unsure.”

#### Sexuality

##### Sexual attraction ratings

Participants rated their sexual attraction to cisgender women, cisgender men, transgender women with a penis, and male cross-dressers on a 10-point slider from 0 (*not at all sexually attracted*) to 10 (*most sexually attracted*). These items assessed conventional attraction to women and men (i.e., gynephilia and androphilia), gynandromorphophilia, and possibly related attraction to male cross-dressers, respectively.

##### Kinsey Scale

The Kinsey scale (Kinsey et al., 1948) is a single-item continuous scale that measures sexual orientation as it is conventionally understood, ranging from 0 (*sexual feelings toward females only*) to 6 (*sexual feelings toward males only*). The item was worded: “Which statement best describes your sexual feelings, overall, during adulthood (after age 18)?”

##### Core Autogynephilia Scale

The Core Autogynephilia Scale (Blanchard, 1989b) is an eight-item measure designed to assess sexual arousal associated with the thought or fantasy of being a woman, possessing a female body, or having specific female physical features (e.g., breasts). An example item reads: “Have you ever become sexually aroused while picturing yourself having a nude female body or with certain features of the female form?” Participants responded to each item dichotomously, indicating whether they had ever been sexually aroused picturing each of the different scenarios. Scores were calculated by summing the number of endorsed items, resulting in a total score ranging from 0 (no items endorsed) to 8 (all items endorsed). Higher scores indicate greater levels of core autogynephilia. Because participants were categorized based on their Core Autogynephilia Scale scores (0–3 = non-autogynephilic; 4–8 = autogynephilic), and because the vast majority of non-autogynephilic participants selected the minimum score (i.e., 0), internal consistency reliability (Cronbach’s *α*) could not be meaningfully computed within each subgroup due to restricted range, low variance, and extreme score skew. Therefore, we report Cronbach’s α for the combined sample of non-autogynephilic and autogynephilic bisexual participants: *α* = 0.94.

##### General Autogynephilia Scale

The General Autogynephilia Scale (Hsu et al., 2015) is a 22-item scale that assesses five facets of autogynephilia, including sexual arousal associated with the thought or fantasy of having specific female anatomy (anatomic autogynephilia), interacting with others as a woman (interpersonal autogynephilia), wearing women’s clothing (transvestic autogynephilia), acting or behaving like a woman (behavioral autogynephilia), and having female physiological functioning (physiologic autogynephilia). Participants were first prompted with the following: “How sexually arousing would you find each of the following activities?” Following the prompt, participants were asked to rate items such as: “Picturing myself with a vagina/vulva” (anatomic autogynephilia), “Picturing myself as a woman being admired by another person” (interpersonal autogynephilia), “Picturing myself wearing a beautiful dress and high-heeled shoes” (transvestic autogynephilia), “Sitting in a feminine way” (behavioral autogynephilia), and “Picturing myself being pregnant” (physiologic autogynephilia). Ratings were made on a five-point scale from 1 (*not at all arousing*) to 5 (*very arousing*). Item ratings were averaged for a range of 1 to 5, with higher scores indicating more general autogynephilia across the five facets that were represented. There was high internal reliability among the non-autogynephilic (*α* = 0.90) and autogynephilic (*α* = 0.94) bisexual men.

##### Paraphilic Interests Scale

The Paraphilic Interests Scale (Hsu et al., 2015) is an 11-item measure that evaluates the number of paraphilic interests as defined by the *Diagnostic and Statistical Manual of Mental Disorders* (American Psychiatric Association, 2013). Items ask about sexual arousal in response to specific behaviors associated with paraphilias such as exhibitionism (items 1–2), fetishism (item 3), voyeurism (items 4–5), frotteurism (item 6), sexual masochism (items 7–8), and sexual sadism (items 9–10). Item 11, which assesses transvestic fetishism (i.e., sexual arousal in response to cross-dressing), was removed from the present study because we were concerned that it would be elevated among the autogynephilic bisexual men by virtue of their autogynephilia. Participants were first prompted with the following: “For each of the following items, please rate how sexually arousing you would find the activity.” Following the prompt, participants were asked to rate their sexual arousal in response to a range of paraphilic behaviors like “being insulted or humiliated by my sexual partner" (sexual masochism). Ratings were made on a five-point scale from 1 (*not at all arousing*) to 5 (*extremely arousing*). Adding one point for each behavior that was rated 3 or higher (at least *somewhat arousing*), scores ranged from 0 to 10 (after removing item 11) with higher scores representing greater endorsement of paraphilic interests. Internal reliability of the measure was good for both the non-autogynephilic (*α* = 0.72) and autogynephilic (*α* = 0.80) bisexual men.

##### Analloeroticism Scale

The Analloeroticism Scale (Bailey & Hsu, 2024) is a 12-item scale assessing the partial or complete lack of sexual interest in others. While individuals with analloeroticism may retain a sex drive, their sexual fantasies and behaviors are not focused on others. Example items include: “During sexual interactions, I am more focused on thinking about myself than about my partner” and “My sexual fantasies always contain people besides myself” (reverse-coded). Participants responded to each question dichotomously with either “agree” (coded as 1) or “disagree” (coded as 0). Adding one point for each question that was answered in the more analloerotic direction, scores ranged from 0 to 12 with higher scores representing more analloeroticism. The scale demonstrated poor internal reliability in both non-autogynephilic (α = 0.42) and autogynephilic (α = 0.52) bisexual men.

##### Sexual Orientation Self-Concept Ambiguity Scale

The Sexual Orientation Self-Concept Ambiguity Scale (Talley & Stevens, 2017) is a 10-item scale that measures the uncertainty of an individual’s sexual orientation self-concept. An example item includes: “My views of my sexual orientation change rapidly or unpredictably.” Ratings were made on a four-point scale from 1 (*strongly disagree*) to 4 (*strongly agree*). Item ratings were averaged for a range of 1 to 4, with higher scores representing more sexual orientation self-concept ambiguity. The scale demonstrated excellent internal reliability among the non-autogynephilic (*α* = 0.93) and autogynephilic bisexual men (*α* = 0.93).

##### Importance of Cross-Dressing in Sexual Contexts

Two items assessed the importance of cross-dressing in sexual contexts. Participants were asked the following two questions: (1) “How important is wearing women’s clothing during masturbation or fantasy?” and (2) “How important is wearing women’s clothing during sex with any person?” Both items were rated on a five-point scale, ranging from 1 (*not at all important*) to 5 (*extremely important*).

##### Sexual and Romantic Partner History

Participants were asked to report the number of sexual and romantic partners they had across their lifetime in each of the following categories: cisgender women, cisgender men, transgender women with a penis, and male cross-dressers.

#### Gender

##### Frequency of Cross-Dressing

Participants were asked the following question: “In the past 12 months, how often have you dressed in women’s clothing for any reason, either in private or in public?” Responses were recorded on a seven-point scale ranging from 1 (*never*) to 7 (*daily*).

##### Childhood Gender Nonconformity Scale

The Childhood Gender Nonconformity Scale (Bailey et al., 1995) is a seven-item scale that measures the degree of self-reported gender nonconformity exhibited during childhood. An example item reads: “As a child, I often felt that I had more in common with girls than boys.” All items were rated on a five-point scale from 1 (*strongly disagree*) to 5 (*strongly agree*). Item ratings were averaged for a range of 1 to 5, with higher scores indicating more childhood gender nonconformity. The internal reliability of this measure was good to excellent across the two groups of non-autogynephilic (*α* = 0.69) and autogynephilic (*α* = 0.72) bisexual men.

##### Adult Gender Nonconformity Scale

The Adult Gender Nonconformity Scale, which was previously called the Continuous Gender Identity Scale (Bailey et al., 1995), is an eight-item measure that in this study assesses the extent to which male respondents feel more feminine than masculine. An example item reads: “In many ways I feel more similar to women than to men.” All items were rated on a five-point scale from 1 (*strongly disagree*) to 5 (*strongly agree*). Item ratings were averaged for a range of 1 to 5, with higher scores indicating more self-reported femininity relative to masculinity, or more adult gender nonconformity. The internal reliability of this measure was acceptable in both non-autogynephilic and autogynephilic bisexual men (*α* = 0.72 and *α* = 0.74, respectively).

##### The Gender Identity/Gender Dysphoria Questionnaire for Adolescents and Adults (GIDYQ-AA)

The GIDYQ-AA (Deogracias et al., 2007), birth-assigned male version, comprises 27 questions related to gender identity and gender dysphoria. These questions were intended to capture diverse aspects of gender identity and gender dysphoria that were experienced by participants in the past 12 months: 13 items had to do with subjective feelings (e.g., “In the past 12 months, have you felt unhappy about being a man?”), 9 items had to do with social activities (e.g., “In the past 12 months, at parties or at other social gatherings, have you presented yourself as a woman?”), three items had to do with somatic experiences (e.g., “In the past 12 months, have you disliked your body because it is male?”), and two items had to do with sociolegal issues (e.g., “In the past 12 months, have you made an effort to change your legal sex?”). Ratings were made in response to each question on a five-point scale from 1 (*never*) to 5 (*always*), referring to the frequency over the past 12 months. The overall score for each participant was calculated by adding up the responses and dividing by the number of items answered. Higher scores indicated more gender dysphoria. The internal reliability of this measure was good to excellent across both groups of non-autogynephilic (α = 0.80) and autogynephilic (*α* = 0.90) bisexual men.

##### Lifetime Gender Dysphoria

Participants were asked whether they had ever experienced gender dysphoria, defined as “discomfort or distress about having a male body or identity.” Responses were dichotomously answered as either “yes” or “no.”

#### Data Analytic Plan

To examine how scores on the various measures differed between autogynephilic and non-autogynephilic bisexual men, we ran a series of Welch’s *t*-tests, which Delacre et al. (2017) recommend using instead of Student’s *t*-tests when sample sizes and variances between groups are unequal. Two variables found to be extremely non-normal, GIDYQ-AA and the General Autogynephilia Scale, were log(*x* + 1) transformed to meet the assumptions of Welch’s *t*-tests. When comparing the frequency and importance of cross-dressing and the number of lifetime sexual and romantic partners, Mann–Whitney *U* tests were used to account for violations of normality assumptions and outliers. A Fisher’s exact test was used to compare the lifetime occurrence of gender dysphoria between non-autogynephilic and autogynephilic bisexual men. To correct for Type 1 error rate when testing group differences across 25 variables, Bonferroni corrections were applied with a significant *p*-value set at 0.05/25 = 0.002.

## Results

### How Common is Autogynephilia Among Bisexual Men?

Figure 1 presents the distribution of scores on the Core Autogynephilia Scale (Blanchard, 1989b) in our bisexual sample. Because there is no established cutoff for whether someone has or does not have autogynephilia, and because the distribution of scores was bimodal, we decided that a midpoint split was most reasonable.

Among bisexual men in our sample recruited from Prolific, 35.0% (89/254) reported scores on the Core Autogynephilia Scale (Blanchard, 1989b) that were greater than or equal to 4. For the sake of comparison, Hsu et al. (2025) found that 14.0% (41/293) of cisgender men, who were also recruited from Prolific but indiscriminately with respect to sexual orientation, scored greater than or equal to 4 on the Core Autogynephilia Scale. Most participants in that sample, specifically 88.40% (259/293), reported that they were heterosexual.

### How Do Autogynephilic and Non-Autogynephilic Bisexual Men Compare on Sexuality?

Table 2 presents the means, standard deviations, effect sizes, and results of independent samples Welch’s *t*-tests and Mann–Whitney U tests comparing autogynephilic and non-autogynephilic bisexual men on their sexual interests and behavior.

**Table 2:** Means, standard deviations, effect sizes, and results of independent samples Welch’s *t*-tests, Mann–Whitney U tests, and Fisher’s exact test comparing non-autogynephilic and autogynephilic bisexual cisgender men on measures of sexuality and gender

Measure	Non-AGP bisexuals (*n* = 165)	AGP bisexuals (*n* = 89)	*t*	*df*	*p*	Cohen’s *d* [95% CI]
	*M* (*SD*)	*M* (*SD*)				
*Sexuality*						
Kinsey Scale	2.39 (1.15)	2.33 (1.09)	0.47	189.08	.640	0.06 [−0.20, 0.32]
Core AGP Scale	0.18 (0.58)	6.27 (1.39)	−39.58	105.09	< .001*	−6.44 [−7.06, −5.82]
General AGP Scale	1.22 (0.34)	2.42 (0.82)	−14.96	118.94	< .001*	−2.30 [−2.63, −1.97]
Paraphilic Interests	2.21 (2.19)	4.00 (2.90)	−5.09	143.49	< .001*	−0.73 [−0.99, −0.46]
Analloeroticism	3.32 (1.81)	3.90 (2.08)	−2.23	159.78	.027	−0.31 [−0.57, −0.05]
SO Ambiguity	1.72 (0.64)	2.08 (0.69)	−4.06	168.25	< .001*	−0.55 [−0.81, −0.29]
*Sexual attraction to:*						
Women	8.35 (1.94)	8.61 (1.90)	−1.01	184.05	.312	−0.13 [−0.39, 0.13]
Men	6.25 (2.77)	6.55 (2.37)	−0.89	205.22	.373	−0.11 [−0.37, 0.15]
Transgender women	4.24 (3.53)	5.16 (3.61)	−1.94	176.74	.054	−0.26 [−0.52, 0.00]
Male cross-dressers	3.15 (3.06)	4.18 (3.23)	−2.47	169.53	.014	−0.33 [−0.59, −0.07]

	*Mdn* (IQR)	*Mdn* (IQR)	*U*	*Z*	*p*	(*r*) [95% CI]
Importance of CD during masturbation	1.0 (1.0–1.0)	1.0 (1.0–1.0)	5992	−4.52	< .001*	−0.15 [−0.23, −0.07]
Importance of CD during sex	1.0 (1.0–1.0)	1.0 (1.0–1.0)	5956	−4.99	< .001*	−0.16 [−0.23, −0.08]
*No. sexual partners:*						
Women	3.0 (1.0–8.8)	3.0 (2.0–7.5)	7170.5	−0.31	.757	−0.02 [−0.17, 0.13]
Men	2.0 (0.0–4.0)	2.0 (0.0–4.0)	7278.0	−0.12	.907	−0.01 [−0.16, 0.14]
Transgender women	0.0 (0.0–0.0)	0.0 (0.0–0.5)	6687.0	−1.72	.086	−0.09 [−0.06, 0.24]
Male cross-dressers	0.0 (0.0–0.0)	0.0 (0.0–1.0)	6695.0	−1.66	.097	−0.09 [−0.06, 0.24]
*No. romantic partners:*						
Women	2.0 (1.0–5.0)	2.0 (1.0–5.0)	7142.5	−0.36	.718	−0.03 [−0.12, 0.18]
Men	0.0 (1.0–5.0)	1.0 (1.0–2.0)	6765.5	−1.12	.262	−0.08 [−0.07, 0.23]
Transgender women	0.0 (0.0–0.0)	0.0 (0.0–0.0)	7247.5	−0.19	.854	−0.01 [−0.14, 0.16]
Male cross-dressers	0.0 (0.0–0.0)	0.0 (0.0–0.0)	6665.0	−2.63	.009	−0.09 [−0.24, −0.06]
*Gender*						
Frequency of CD	1.0 (1.0–1.0)	1.0 (1.0–2.0)	5590	−4.86	< .001*	−0.20 [−0.29, −0.11]

	M (SD)	M (SD)	t	df	p	Cohen’s d [95% CI]
GIDYQ-AA	1.20 (0.25)	1.61 (0.65)	−6.12	109.29	< .001*	−0.98 [−1.25, −0.71]
Child GN	1.95 (0.65)	2.29 (0.72)	−3.83	166.77	< .001*	−0.52 [−0.78, −0.26]
Adult GN	2.07 (0.63)	2.79 (0.68)	−8.24	167.73	< .001*	−1.11 [−1.39, −0.84]

Uncorrected *p* values are reported for data transparency. To correct for multiple comparisons (25 tests), a Bonferroni-corrected significance threshold was applied: .05/25 = .002 (i.e., adjusted alpha level set at *p* < .002). Asterisks indicate statistical significance based on this corrected threshold.

Log(*x* + 1) transformations were applied to the General AGP Scale and GIDYQ-AA to meet normality assumptions for Welch’s *t*-tests.

From top to bottom, absolute ranges for measures (excluding those for numbers of sexual and romantic partners) were 0–6, 0–8, 1–5, 0–10, 0–12, 1–4, 0–10, 0–10, 0–10, 0–10, 1–5, 1–5, 1–7, 1–5, 1–5, and 1–5.

*M* = mean; *SD* = standard deviation; *Mdn* = median; *IQR* = interquartile range; AGP = autogynephilia; SO = Sexual Orientation; CD = Cross-dressing; GIDYQ-AA = Gender Identity/Gender Dysphoria Questionnaire for Adolescents and Adults; GN = Gender Nonconformity.

As predicted, relative to non-autogynephilic bisexual men, autogynephilic bisexual men reported a greater importance of cross-dressing to their solo masturbation, sexual fantasizing, as well as partnered sex. They also scored higher than non-autogynephilic bisexual men on the two measures of autogynephilia, paraphilic interests, and sexual orientation uncertainty. Analloeroticism was significantly higher in autogynephilic bisexual men than non-autogynephilic men at the conventional *α* = 0.05 (*p* = 0.019), but this effect did not remain significant after adjusting for multiple comparisons. No significant differences in scores on the Kinsey scale (Kinsey et al., 1948), ratings of sexual attraction to cisgender women (i.e., gynephilia), cisgender men (i.e., androphilia), or transgender women with a penis (i.e., gynandromorphophilia), and numbers of lifetime sexual or romantic partners who were cisgender women, cisgender men, or transgender women with a penis were observed between autogynephilic and non-autogynephilic bisexuals. Autogynephilic bisexual men reported greater attraction to male cross-dressers and more romantic partners who were male cross-dressers than non-autogynephilic bisexual men (*ps* < 0.05), but these effects did not survive correction for multiple comparisons.

### How Do Autogynephilic and Non-Autogynephilic Bisexual Men Compare on Gender?

Table 2 presents the means, standard deviations, effect sizes, and results of independent samples Welch’s* t*-tests and the Fisher’s exact test comparing differences between autogynephilic and non-autogynephilic bisexual men on their gender-related experiences and distress.

As predicted, autogynephilic bisexual men reported a greater frequency of cross-dressing and scored higher than non-autogynephilic bisexual men on childhood and adult gender nonconformity, as well as gender dysphoria. A Fisher’s exact test revealed a statistically significant association between autogynephilia and lifetime occurrence of gender dysphoria among bisexual men, *p* = 0.00199. Specifically, lifetime occurrence of gender dysphoria was reported by 11.2% of autogynephilic bisexual men (10 out of 89) and 1.2% of non-autogynephilic bisexual men (3 out of 165). The odds of reporting gender dysphoria were significantly higher among autogynephilic bisexual men compared to non-autogynephilic bisexual men, odds ratio = 6.84, 95% CI = [1.83, 25.54].

## Discussion

This study was the first to examine the prevalence of autogynephilia in a sample of bisexual cisgender men. More than one-third (35%) of bisexual men in our sample reported autogynephilia. Consistent with expectations, autogynephilic bisexuals reported more uncertainty about their sexual orientation, elevated paraphilic interests, and less sexual interest in others, although this latter finding failed to reach the adjusted significance threshold. Contrary to our predictions, autogynephilic bisexuals were not significantly less sexually attracted to cisgender men, or significantly more sexually attracted to transgender women with a penis relative to non-autogynephilic bisexuals. Likewise, no significant differences in the numbers of lifetime sexual or romantic partners who were cisgender men or transgender women with a penis were observed. Consistent with predicted effects, autogynephilic bisexuals reported greater sexual attraction to male cross-dressers and more romantic relationships with male cross-dressers, but these effects were not significant after correcting for multiple comparisons. As expected, autogynephilic bisexuals demonstrated significantly greater discomfort with their gender, as shown by higher scores on a validated measure of gender dysphoria and a greater lifetime occurrence of gender dysphoria. Finally, autogynephilic bisexuals indicated greater self-perceived gender nonconformity in both childhood and adulthood, relative to non-autogynephilic bisexuals.

How much do we believe that 35% of bisexual men are autogynephilic? We think that it is certainly plausible, considering that up to 3% of men in a nationally representative sample reported erotic cross-dressing (Långström & Zucker, 2005), and at least 1% of men identify as bisexual (Wilson et al., 2020). However, it is possible that our sample attracted a larger proportion of autogynephilic bisexual men than is true of the bisexual male population at large. This situation may have occurred because the advertisements that we used to recruit for this study mentioned a focus on gender, sexuality, and identity. However, this situation also seems unlikely based on the results of another study, where only 14% of cisgender men recruited indiscriminately with respect to sexual orientation and using the same recruitment source reported autogynephilia based on our cutoff scores (Hsu et al., 2025), and those advertisements similarly mentioned a focus on gender, sexuality, and identity. While it could be argued that this prevalence of 14% is inflated relative to past estimates such as that of 3% from Långström and Zucker (2005), bisexual men are still more likely to be autogynephilic than the general male population, at least when comparing on Prolific and using our criteria for determining who is autogynephilic or not. Furthermore, the explicitly mentioned requirement that participants not identify as either cross-dressers or transfeminine would be expected to decrease rather than increase the probability of bisexual men with autogynephilia participating in this study. Given the lack of understanding of autogynephilia, and the stigma and discrimination directed toward those who experience it (Lawrence, 2013; Serano, 2020), we would also expect that some bisexual men who are in fact autogynephilic may not disclose these feelings.

Another possibility unrelated to our recruitment approach is that elevated scores on the Core Autogynephilia Scale (Blanchard, 1989b) reflected measurement error, such as careless responding, misinterpretation of items, intentional efforts to distort data, or other types of spurious responding. In contrast to this possibility, high mean scores observed on both the Core Autogynephilia Scale and the General Autogynephilia Scale (Hsu et al., 2015) indicate consistent endorsement across two different scales of autogynephilia (see Table 2). Perhaps the most compelling evidence of genuine autogynephilia among some bisexuals in the present sample relates to their consistent pattern of responding on relevant sexuality and gender variables that indicated differences from non-autogynephilic bisexuals. As predicted, autogynephilic bisexuals reported more frequent cross-dressing and greater importance of cross-dressing during masturbation and sex, as well as more uncertainty about their sexual orientation, paraphilic interests, analloeroticism, attraction to and relationships with male cross-dressers, gender dysphoria, and gender nonconformity. It seems unlikely that spurious responding alone could account for the pattern of results observed. Of course, it remains possible that the prevalence of autogynephilia observed in our bisexual sample was to some degree inflated due to measurement error.

What is the relationship between bisexual identity and autogynephilia among autogynephilic bisexuals in our sample? Blanchard’s (1989b) explanation is that autogynephilia provides the motivation for men to become sexually aroused by sexual fantasies and behavior involving men because imagining themselves as a woman during these sexual fantasies and behavior with men is consistent with their autogynephilic desire to be more like a woman. In other words, because most women tend to sexually fantasize and have sex with men, some autogynephilic men might be especially motivated to do the same. Moreover, fantasy and sexual or romantic intimacy involving men provides the opportunity to be treated as a woman, or more broadly, as feminine and sexually receptive. Sexual and romantic encounters with men, especially when cross-dressed or adopting a submissive role, can provide powerful interpersonal autogynephilic experiences. For instance, in such encounters an autogynephilic bisexual man may be called by a feminine name, may take a submissive role in physical contact (e.g., when kissing, holding hands, or cuddling), and enjoy being penetrated and sexually dominated. Experiences of this type can be found online, such as in this entry by a heterosexually married man on r/bisexual: “[I have] always been attracted to women [and] men don’t attract me physically [but] I have lots of fetishes like cross-dressing, sissy porn, and think I would enjoy being penetrated by a man and pleasuring a man. But I don’t think I could be a top to a man, I only want to be a bottom to a man” (LizardCurious, 2022). This elevated interest in sexual fantasies and behaviors involving men, combined with conventional attraction to women, could conceivably lead autogynephilic men to identify as and believe that they are bisexual. It seems likely that this pathway to bisexual identity accounts for at least some proportion of autogynephilic bisexual men in our sample.

There are other possibilities, however. An autogynephilic man might additionally or alternatively identify as bisexual or queer because he interprets his autogynephilic sexual interests or activities as non-heteronormative. For instance, some common expressions of autogynephilia like cross-dressing, wearing women’s lingerie, being anally penetrated (e.g., pegging), and preferring non-penetrative sex or less dominant sexual roles that might include explicitly submissive or masochistic play with female partners might be interpreted as queer or non-heteronormative, leading an autogynephilic man to identify or feel as if he is bisexual. Furthermore, given that autogynephilia is associated with gynandromorphophilia (Hsu et al., 2016; Rosenthal et al., 2017), preferential attraction to transgender women with a penis and other feminized birth-assigned males, could be informing bisexual identity in some autogynephilic men. Indeed, gynandromorphophilic men appear to have elevated rates of bisexual identity due to their belief that sexual attraction to individuals with both male and female physical characteristics implies bisexual attraction. It is also possible that some autogynephilic men are bisexual in the conventional sense of being sexually attracted to both cisgender women and men. However, because the best-established base rates of bisexuality (Wilson et al., 2020) and autogynephilia (Långström & Zucker, 2005) are low in the general population, the two independently co-occurring would seem unlikely. Future research should seek to disentangle these differing reasons for why autogynephilic bisexual men might identify as bisexual.

Autogynephilic bisexual men demonstrated a range of sexual and gender-related traits not generally associated with male bisexuality, as evidenced by their higher scores relative to non-autogynephilic bisexual men. Specifically, they demonstrated elevated gender dysphoria, and less sexual interest in other people, although this latter finding was not significant after correcting for multiple comparisons. In addition to possibly influencing their sexual behavior, including more uncertainty about their sexual orientation, these traits may influence other aspects of their sexuality, relationship functioning, and emotional well-being. Future research should examine these possibilities.

To the extent that autogynephilic bisexual men experience sexual attraction to men only in the context of imagining themselves as women, they may question whether their sexual attraction is consistent with a true bisexual orientation. Whereas traditional explanations of sexual confusion in bisexual men posit invalidating effects of bisexual erasure (see e.g., Balsam & Mohr, 2007), it is possible that autogynephilia is also a potent source of sexual confusion. Future research could examine whether psychoeducation regarding autogynephilia reduces sexual orientation uncertainty. It is notable that only 18% of autogynephilic bisexual men in our sample had heard of the phenomenon, suggesting a lack of education and awareness about autogynephilia. It would be important to examine whether it is autogynephilia specifically, its associated paraphilic interests or analloeroticism, or some combination of these factors that contributes to sexual confusion or sexual orientation uncertainty. Like autogynephilia, paraphilic interests and analloeroticism are poorly understood in the wider culture, and they may also lead to confusion about sexual orientation and someone identifying as bisexual, asexual, queer, or something other than heterosexual (Blanchard, 1991; Hsu & Bailey, 2022; Lawrence, 2009).

### Limitations and Future Directions

The present findings should be interpreted cautiously due to important limitations. First, our estimate of autogynephilia prevalence in bisexual men was based on a convenience sample of bisexual men who were recruited from Prolific and who may have differed in important respects from the bisexual male population at large. Our sample was mostly White/Caucasian, highly educated, non-religious, and politically liberal. Perhaps owing to the advertising and online administration, our study may have attracted a greater proportion of autogynephilic bisexual men. Future studies should seek to examine the prevalence of autogynephilia in bisexual men recruited via other online avenues (e.g., dating or hook-up apps for men who have sex with men, LGBTQIA2S social media platforms).

Second, findings in this study were based entirely on self-report. Due to the heightened rate of sexual orientation uncertainty among the autogynephilic bisexual men in our sample, as well as the different possibilities that exist for autogynephilic men to conceive of themselves as bisexual without experiencing conventional sexual attraction to both cisgender women and men (e.g., autogynephilic bisexuality, frequently co-occurring gynandromorphophilia), it would be important for future research to supplement self-report measures of autogynephilia and especially bisexual phenomena with more objective measures that are not subject to bias. For example, autogynephilic bisexual men who lack insight into their motivations for identifying as bisexual could be tested in the laboratory for whether they display bisexual arousal patterns that are more consistent with autogynephilic bisexuality, gynandromorphophilia, or bisexual orientation as it is conventionally understood. While autogynephilic bisexual men in this study did not differ from non-autogynephilic bisexual men on the Kinsey scale, ratings of sexual attraction to cisgender women and men, or numbers of lifetime sexual or romantic partners who were cisgender women and men, their self-reports could be influenced by their bisexual identity or beliefs that they are bisexual. Psychophysiological indices of sexual arousal such as genital arousal, eye-tracking, or pupil dilation would provide objective evidence for or against the proposition that autogynephilic bisexual men are bisexual due to autogynephilia, gynandromorphophilia, conventional attraction to men and women, or other reasons. A study that measured the genital arousal of autogynephilic male cross-dressers in response to different sexual stimuli found no difference between their sexual arousal patterns and those of gynandromorphophilic men (Hsu et al., 2017). However, only 16 autogynephilic participants in that study had usable genital arousal data, four of whom identified as bisexual, which precludes strong conclusions.

Finally, the present study only examined the prevalence of autogynephilia among bisexual men. It is possible that autogynephilia is a relatively common contributor to bisexual phenomena even among men who identify as mostly heterosexual (Savin-Williams & Vrangalova, 2013) or who adopt sexual identities such as pansexual or queer (Morandini et al., 2017). Mostly heterosexual, pansexual, or queer men may similarly come to believe that they are not completely heterosexual if their autogynephilic feelings or fantasies involve men, imagining themselves as a woman with a man, or engaging in behaviors often associated with queer men (e.g., cross-dressing, anal penetration). Future research should seek to examine the representation of autogynephilia among non-heterosexual populations besides bisexual men.

### Implications

The present findings suggest that acknowledging the diversity of bisexual men, in this case autogynephilic bisexual men, may assist basic and applied research, advocacy, and social and healthcare support for bisexual men. First, it seems that knowing whether a bisexual man is autogynephilic has predictive value in understanding his sexual and emotional struggles (e.g., sexual orientation uncertainty, gender dysphoria, and anomalous sexual interests that might cause further confusion or distress). Second, information on the unique and shared experiences between autogynephilic and non-autogynephilic bisexual men will allow for more tailored clinical and public health interventions. Third, our findings highlight the need for additional research, support, and advocacy to improve the lives of autogynephilic bisexuals, whose experiences differ from those of other bisexuals but are largely invisible at present. Meaningful changes might include a focus on destigmatizing autogynephilic bisexuality and emphasizing that this pathway to bisexuality is not better or worse than other pathways. Fourth, both basic and applied research on bisexual men should move toward acknowledging the heterogeneity within this group by developing a formal typology of male bisexuality, assessing relative prevalence of different types of male bisexuality, and designing research informed by these distinct types of bisexuality in men.

### Conclusions

The present study found that a little more than one-third of bisexual men from a large online convenience sample were autogynephilic, and these autogynephilic bisexual men demonstrated a unique profile of sexual interests and gender experiences compared with non-autogynephilic bisexual men. Autogynephilic bisexual men demonstrated higher rates of paraphilic interests, uncertainty about their sexual orientation, gender nonconformity, and gender dysphoria. It would behoove researchers and clinicians to acknowledge this largely hidden subgroup of bisexual men.

## Data Availability

Available upon email request to the corresponding author.

## Code Availability

Available upon email request to the corresponding author.
